# Cases Illustrative of the Pathology and Surgical Treatment of Some of the Diseases of the Testicle; with Observations

**Published:** 1826-12

**Authors:** B. C. Brodie


### DISEASES OF THE TESTICLE.

*Cases illustrative of the Pathology and Surgical Treatment of some of the Diseases of the Testicle; with Observations.* By B. C. BRODIE, F.R.S. &c. &c.

(Continued from page 312.)

### IV. *Encysted Hydrocele.*

IN some cases, in consequence of inflammation at some former period, the opposite surfaces of the tunica vaginalis adhere throughout a great part of their extent; while a serous fluid is collected in that part of the tunica vaginalis in which the adhesions are wanting, so as to form what may be regarded as a partial hydrocele.

In other cases we find a hydrocele with a contraction in the centre, giving the tumor somewhat of the form of an hour-glass. In general, at the contracted part there is a channel of communication, by which the fluid may be made to pass from one portion of the tumor into the other; but occasionally it happens that the contraction in the centre is so complete that this channel of communication is wanting, and then, although the upper and lower portions of the tumor are connected with each other, their cavities are distinct, so that, when one of them is emptied by means of the trocar, the other remains distended with fluid.

Now, such tumors are liable to be confounded with the tumor of the encysted hydrocele, although really and essentially different from it. The latter disease is, in fact, altogether independent of the tunica vaginalis, and bears a much closer resemblance to a common encysted tumor than to a proper hydrocele.

Case XVI. *Dissection of an Encysted Hydrocele of the Spermatic Chord.*

A young man, who laboured under an encysted hydrocele of the spermatic chord, was admitted into St. George's Hospital, under the care of the physician, on account of another disease, of which he died in February 1814.

On examining the encysted hydrocele after death, it was found to be a distinct cyst, having no connexion with the tunica vaginalis. The cyst was composed of a thin transparent membrane, containing a colourless fluid, and lay in the cellular texture of the spermatic chord, between the spermatic artery and vein and the vas deferens. It was of the size of a small walnut, perfectly moveable upward and downward, in consequence of it having only a very loose adhesion to the surrounding parts.

Case XVII. *Dissection of an Encysted Hydrocele of the Epididymis.*

An elderly gentleman had for many years a tumor connected with one testicle, which, however, gave him neither pain nor inconvenience. It appeared as if the testicle on this side was divided into two lobes, each as large as the testicle itself is under ordinary circumstances.

In April, 1821, the patient died in consequence of an extensive abscess, which had formed in the substance of the prostate gland. On examining the body after death, it was ascertained that the tumor of the testicle was formed by a membranous cyst, containing a watery fluid, and attached to the epididymis. The tumor was covered by the tunica vaginalis, lying between it and the epididymis, and connected to them only by a very fine cellular texture.

Case XVIII. *Dissection of an Encysted Hydrocele of the Testicle.*

A man, who died in St. George's Hospital of another disease, was discovered, after death, to have had encysted hydrocele of one testicle.

The cyst was composed of a thin membrane, containing a colourless fluid, and was of about the size of a walnut: it was attached to the anterior part of the testicle, below the epididymis. The inner layer of the tunica vaginalis was reflected over one side of the cyst, while the cyst on the other side rested on the fibrous membrane of the tunica albuginea, by which it was in consequence separated from the glandular structure of the testicle.

It was evident that the cyst had been originally formed between the tunica albuginea and the inner layer of the tunica vaginalis, and that, in its growth, it had separated from each other these two membranes, which under natural circumstances are so closely adherent to each other.

The encysted hydrocele of the spermatic chord, being very loosely connected to the surrounding parts, forms a very moveable tumor, which may be made to ascend into the groin or to descend into the scrotum, accordingly as pressure is applied to it from above or below. When of a small size, it may be made to enter the abdominal ring, and hence is liable to be mistaken by a superficial observer for an inguinal hernia. It is, for the most part, unattended by pain, and not productive of actual inconvenience to the patient, except such as arises from its bulk. On the other hand, the encysted hydrocele of the testicle or epididymis, being bound down by the internal layer of the tunica vaginalis, is necessarily fixed in its situation, giving the testicle the appearance of being double or lobulated. In some cases the patient is very little molested by it; but in other cases I have observed that he experiences a good deal of pain, in addition to the inconvenience which is caused by the bulk of the tumor. In young persons, the encysted hydrocele, even when of a considerable size, admits of being cured by the application of stimulating lotions to the skin. In the adult, however, I have never known this method of treatment to succeed; but a cure may be effected by passing a seton through the cyst, or by making an opening into it, and dressing its cavity with lint.

The following cases are intended to illustrate the foregoing observations:

Case XIX. *Encysted Hydrocele of the Spermatic Chord in a Boy, cured by the application of a Stimulating Lotion.*

In September, 1818, a gentleman brought me his son, a boy of ten years of age, saying that he had a hernia, for which he had been recommended to wear a truss. The supposed hernia was a globular tumor, loosely connected to the spermatic chord, capable of being moved upwards as high as the groin, and downwards as low as the scrotum. It could be made to pass through the external abdominal ring: it did not, however, then enter the abdomen, but remained in the inguinal canal, making a projection behind the tendon of the external oblique muscle. I prescribed a lotion of the muriate of ammonia, dissolved in distilled vinegar and alcohol. This was kept constantly applied for two or three months, at the end of which time the tumor had entirely disappeared. There has been no return of the tumor up to the present period, (August 1826.)

Case XX. *Encysted Hydrocele of the Spermatic Chord in an Infant, cured by the application of a Stimulating Lotion.*

In the summer of 1820, I was consulted, with Mr. Hillman, of Argyll-street, respecting a little boy, an infant a few months old, whose parents had supposed him to labour under a hernia.

On examination, I discovered a tumor connected with the spermatic chord, apparently containing fluid, and of the size of a large walnut. The tumor was distinct from the testicle below, and from the abdominal ring above, and the bulk of it was not increased when the child cried. A puncture having been made with a lancet, a considerable quantity of fluid escaped, and the tumor was no longer perceptible. The wound made by the lancet healed; but in a short time the tumor was again discovered, and became of as large a size as before the puncture. I then advised the application of a strong solution of the muriate of ammonia, and under this treatment the tumor disappeared in the course of a few months.

Case XXI. *Encysted Hydrocele of the Spermatic Chord in an Adult, cured by the introduction of a Seton.*

A gentleman, in the year 1813, consulted me respecting a small hydrocele of the tunica vaginalis of the right testicle. The hydrocele was injected, and he was cured.

In the year 1817, he consulted me respecting a tumor in the right groin, which he had first noticed soon after the cure of the hydrocele. The tumor was of the size of a walnut, and manifestly contained fluid. It was connected with the spermatic chord, and capable of being moved upwards and downwards in the chord. It could even be made to enter the external abdominal ring, and hence some practitioners, who had been consulted, had been led to regard the case as one of hernia. More careful examination, however, rendered the difference between this tumor and that of hernia sufficiently distinct. When it had been made to pass through the abdominal ring, it nevertheless did not enter the abdomen, but remained just perceptible behind the tendon of the external oblique muscle; and, when it was made to descend into the scrotum, the spermatic chord was left uncovered between the tumor and the abdominal ring. The tumor varied in size, and was observed to be diminished after two or three days of rest.

A solution of muriate of ammonia having been applied for some time without any evident advantage, I performed the following operation. The integuments over it having been divided with a sharp-pointed bistoury, the tumor presented itself as a transparent cyst, containing a colourless fluid. I then passed through the cyst, from below upwards, a straight flat needle, carrying a few threads of silk. The fluid of course escaped, and the cyst collapsed. The silk was allowed to remain, forming a small seton, which was withdrawn at the end of a week. The wound immediately healed, and nothing remained of the disease except a very small solid substance, which was only just perceptible, and which soon disappeared.

The patient has continued without any recurrence of the disease up to the present time, (August 1826.)

Case XXII. *Encysted Hydrocele of the Spermatic Chord in an Adult, cured by the introduction of a Seton.*

April 4th, 1826.—A gentleman consulted me respecting a tumor in the left groin containing fluid, and of the size of a double walnut. It was connected with the spermatic chord, lying on the anterior part of it, and so far moveable that it admitted of being pushed downwards into the scrotum, and upwards as high as the abdominal ring; which, however, it was considerably too large to enter.

The tumor was punctured with a trocar, and a transparent colourless fluid escaped, which, being exposed to heat in a spoon over the flame of a candle, proved to contain no coagulable matter, and evaporated, leaving a residuum so small in quantity that it was barely perceptible.* A lotion, containing distilled vinegar, spirit, and muriate of ammonia, was prescribed, to be constantly applied to the part.

10th.—The fluid had collected again, and the tumor was as large as ever.

13th.—A straight flat needle, carrying a double silk thread, was passed through the centre of the tumor. The silk threads were allowed to remain during four days, and then withdrawn. In a few days after the removal of the seton, suppuration ceased. A small hard tubercle remained, indicating the part at which the encysted hydrocele had been situated; and this gradually disappeared.

Case XXIII. *Encysted Hydrocele of the Testicle, cured by the introduction of a Seton.*

A. B., a young man, in the year 1812, observed a small tumor connected with the right testicle. The tumor gradually increased during the three following years, after which it remained nearly stationary. In April, 1818, he consulted me respecting it. At this time there was a tumor attached to the upper and outer part of the right testicle, nearly as large as the testicle itself, and evidently containing fluid.

On the 16th of April, I punctured the tumor with a lancet, and about a quarter of an ounce of watery fluid escaped. I then introduced an eye-probe, carrying a few threads of silk into the puncture, and, having made a second puncture on the opposite side, I passed the probe through the counter opening, and thus drew the silk threads, as a seton, through the cavity of the cyst.† A very

* The fluid in this case differed from that of common hydrocele, which entirely coagulates on the application of heat. Whether the fluid of encysted hydrocele is always free from coagulable matter, as it was in this instance, has not (as far as I know) been ascertained.

† Although it succeeded perfectly in this instance, my experience in many other cases has satisfied me that this is not the most convenient method of performing the operation; and that it is better to employ a single instrument, such as a flat needle, for the double purpose of puncturing the cyst and introducing the seton.

slight degree of inflammation followed the operation. On the 18th of April, the seton having slipped out, it was re-introduced, and allowed to remain three days longer, when it was altogether withdrawn. Suppuration had taken place in the wound round the seton. The discharge of pus gradually lessened, and in a few days there was only a small solid tubercle left in the place of the original disease; and this had almost entirely disappeared when I again saw my patient, on the 9th of May following.

As the cyst of the true encysted hydrocele is proved by dissection to be altogether a new formation, independent of the tunica vaginalis, it cannot be a matter of surprise that a disease corresponding to it should sometimes be met with in the groin of the female sex. I have lately seen a lady who has a cyst in one groin, immediately below the abdominal ring, as large as a hen's egg, containing fluid, to a certain degree moveable, and so entirely free from pain that the discovery of it was almost accidental. The following case affords another example of such a tumor occurring in the female sex.

Case XXIV. *Tumor in the Female Groin, corresponding to the Encysted Hydrocele, and cured by an Operation.*

In September, 1824, I was consulted, in conjunction with Mr. Freeman of Spring-gardens, by a female patient with a tumor in the groin, of the size of a pigeon's egg, somewhat moveable in the cellular texture, and containing fluid. The examination of the tumor occasioned pain, extending from the groin into the abdomen; from which circumstance we were led to suspect that it was attached to the termination of the round ligament of the uterus, where it passes out through the abdominal ring to be gradually lost in the labium pudendi. The case had been supposed, by a gentleman who had been consulted previously, to be one of irreducible hernia, but a careful investigation of it satisfied us that this opinion was erroneous.

An incision having been made in the groin, so as to expose the surface of the cyst, the latter was laid open, and a serous fluid escaped from its cavity. A portion of the cyst was then removed with the knife, and some lint was introduced into that which remained. Suppuration having taken place, the wound was dressed daily to the bottom, until it was filled up with granulations. In

If we use a lancet for the first purpose, and a probe for the second, it often will happen that, in consequence of the thin membrane of the cyst becoming immediately collapsed, the opening in it ceases to correspond with the opening in the skin; and then the probe, instead of taking its proper course, penetrates only into the loose cellular texture of the scrotum. The latter affords so little resistance, that the surgeon may easily be led into the belief that the probe is being passed through the cavity of the cyst, when in reality it is sliding through the parts external to it. The error is afterwards demonstrated by the failure of the operation and the return of the hydrocele.

about a month it was healed, and there were no perceptible remains of the tumor.

As the encysted hydrocele is not a disease from which any ill consequences are to be apprehended, there is no reason for the patient being subjected to the inconvenience of an operation for its relief, until it begins, in some way or other, to be a source of actual inconvenience; and, on the other hand, whenever this period arrives, there is no reason why an operation should not be at once resorted to. It is right, however, that I should not pass over unnoticed one case which came under my observation, in which the exposure of the cavity of an encysted hydrocele of the testicle was followed by severe inflammation of the testicle itself. At the end of a week, when this began to subside, the patient was seized with pain in the head, and other symptoms of determination of blood to the brain, requiring the free use of the lancet. These symptoms having abated, he became affected with cough and pain in the chest, which also required loss of blood from the arm. At the end of a month, the wound in the scrotum having been for some time healed, and the patient apparently convalescent, so as to undertake a journey of some miles into the country, there were symptoms which seemed to indicate a sudden effusion of fluid into the cavity of the chest, and which were followed by death. Here there was a disturbed state of the constitution beginning with inflammation of the testicle, but certainly not to be ascribed to the operation, so much as to a peculiar condition of the general system at the time of its being performed. In all probability, any slight accidental injury, exposure to cold, and various other causes, of which it might have been impossible to take cognizance, would, under the same circumstances, have been followed by a similar train of symptoms, leading to the same result.

I shall conclude this section with a brief account of two cases, which must be regarded as being rare, as I have never met with any others similar to them. I leave it to future inquirers to explain the exact nature of the disease, merely observing that they are not improperly introduced in this place, as such cases must be liable to be confounded with those of encysted hydrocele.

Case XXV. *The Cavity of the Tunica Vaginalis divided by adhesion into two Parts, in one of which was a Collection of a peculiar Fluid.*

A man was admitted into St. George's Hospital, on account of a disease in his lungs, in whom a tumor was attached to the left testicle, giving it the appearance of being double or lobulated. He died of the pulmonic affection, and I did not lose the opportunity of examining the tumor of the testicle. There was an adhesion of the opposite surfaces of the tunica vaginalis, below the epididymis and nearly parallel to it, by which the cavity formed by that membrane was divided into two parts. That part which belonged to the testicle was lubricated by a little moisture, as usual; while that which corresponded to the epididymis was distended with about a quarter of an ounce of fluid. The fluid was of a dingy yellow colour, wholly different from pus, and depositing its colouring matter in the form of a yellow sediment, when allowed to remain at rest. The tunica vaginalis bore no marks of inflammation.

Case XXVI.—Of the second of the two cases to which I have alluded, unfortunately I have no written notes. The subject of it presented himself as an out-patient at the hospital, on account of a tumor, containing fluid, attached to one testicle, nearly as large as the gland itself, and without any evidence of inflammation. I made a free opening into it with a lancet, and immediately the cyst collapsed, having discharged a dingy yellow fluid, apparently similar to that which was found to exist in the preceding case. Some degree of inflammation of the cellular texture of the scrotum immediately adjoining the puncture, followed the operation. The puncture continued to discharge fluid, and, when the patient returned to the hospital at the end of a few days, there was a fistulous sinus, surrounded by a gristly induration, opening on the surface of the scrotum, through which a probe could be introduced so as to come in contact with the testicle. The sinus seemed to be gradually healing, but I lost sight of the patient before his cure was completed.

[To be continued.]

